# CXCL16 Promotes Gastric Cancer Tumorigenesis via ADAM10-Dependent CXCL16/CXCR6 Axis and Activates Akt and MAPK Signaling Pathways

**DOI:** 10.7150/ijbs.57826

**Published:** 2021-07-05

**Authors:** Jing Han, Runjia Fu, Cong Chen, Xiaojing Cheng, Ting Guo, Longtao Huangfu, Xiaomei Li, Hong Du, Xiaofang Xing, Jiafu Ji

**Affiliations:** 1 Department of Gastrointestinal Cancer Translational Research Laboratory, Key Laboratory of Carcinogenesis and Translational Research (Ministry of Education), Peking University Cancer Hospital & Beijing Institute For Cancer Research, Fu-Cheng Road, Beijing, China.; 2 Department of Oncology, The Second Hospital, Cheeloo College of Medicine, Shandong University, Jinan, 250033, China.; 3 Department of Gastrointestinal Surgery, Key Laboratory of Carcinogenesis and Translational Research (Ministry of Education), Peking University Cancer Hospital & Institute, Beijing, China.

**Keywords:** gastric cancer, CXCL16, ADAM10, tumorigenesis

## Abstract

Abnormal expression of CXC motif chemokine ligand 16 (CXCL16) has been demonstrated to be associated with tumor progression and metastasis, served as a prognostic factor in many cancers, with higher relative expression behaving as a marker of tumor progression. However, its role and mechanisms underlying progression and metastasis of gastric cancer (GC) are yet to be elucidated. In our investigation, public datasets and human GC tissue samples were used to determine the CXCL16 expression levels. Our results revealed that CXCL16 was upregulated in GC. The high expression CXCL16 in GC was significantly associated with histologic poor differentiation and pTNM staging. And high CXCL16 was positively correlated with the poor survival of GC patients. Gain-and loss-of-function experiments were employed to investigate the biological role of CXCL16 in proliferation and migration both *in vitro* and* in vivo*. Mechanically, Gene set enrichment analysis (GSEA) revealed that the epithelial‑mesenchymal transition (EMT), Akt and MAPK signal pathway related genes were significantly enriched in the high CXCL16 group, which was confirmed by western blot. Moreover, overexpression CXCL16 promoted the disintegrin and metalloproteases (ADAM10) and the CXC motif chemokine receptor 6 (CXCR6) expression, which mediated the CXCL16/CXCR6 positive feedback loop in GC, with activating Akt and MAPK signaling pathways. Knocking down ADAM10 would interrupted the CXCL16/CXCR6 axis in the carcinogenesis and progression of GC. In conclusion, our findings offered insights into that CXCL16 promoted GC tumorigenesis by enhancing ADAM10-dependent CXCL16/CXCR6 axis activation.

## Introduction

GC is one of the major malignancies in the world and the second leading cause of cancer-related death worldwide 1. GC still constitutes a huge health threat in Asia, such as in China and Japan 2, 3. Despite surgery as the predominant treatment for GC, many patients develop advanced stage disease, or experience postsurgical disease relapse or metastasis, which make their prognoses even worse 4. Comprehensive treatment of advanced GC remains unsatisfactory. For GC tumorigenesis is a multistep and multifactorial process that is associated with several genetic and molecular alterations, there is less effective approach to predict tumorigenesis and prevent recurrence. Hence, it is urgent to unveil the molecular mechanisms underlying tumorigenesis and progression, and to develop prognostic and therapeutic strategies.

In cancer biology, accumulating reports demonstrated that chemokines were associated with tumorigenesis, progression, metastasis and angiogenesis, as well as the tumor microenvironment 5-8. The chemokine signaling networks in tumor could be an ideal cancer therapy target as well as potential agents for immunotherapy, reflecting their multifaceted role in the development and progression of cancer 9. CXC chemokines are widely expressed in digestive cancers and correlated with poor prognosis 10. Among various these CXC chemokines, CXCL16 has been described in both transmembrane CXCL16 (TM-CXCL16) and soluble forms (sCXCL16). Membrane-bound CXCL16 can be released as the soluble form upon digestion by ADAM10. CXCL16's orphan ligand is CXCR6 11. The CXCL16/CXCR6 signaling axis has been reported involved in several kinds of tumor and in multiple signaling pathways in malignant cells, suggesting that CXCL16/CXCR6 axis is a critical role in tumor tumorigenesis and progression 12-14. However, the expression of CXCL16 correlate with GC patient survival is understudied. Interestingly, ADAM10, a member of transmembrane metalloproteases, responsible for cleaving CXCL16, has also been implicated as a diagnostic and prognostic marker for multiple tumors and associated with poor prognosis in GC 15-18.

Previously, we conducted a gene expression profiling on 198 cancer and paired adjacent normal tissues from Chinese GC patients by RNA array with 40,914 transcripts 19. CXCL16 was one of genes that significantly upregulated in the tumor tissue. In the present study, we investigated the association between CXCL16 expression and clinicopathologic parameters in GC patients as well as it in predicting prognosis. We determined the effect of CXCL16 on GC tumorigenesis through overexpression and knockdown in GC cells *in vivo* and *in vitro*. Moreover, we also further explored how ADAM10 mediated CXCL16 the underlying mechanism for tumorigenesis, thus provided a novel insight into the pathology and treatment of GC.

## Materials and methods

### Patient samples

A total of 149 GC tissues were obtained from patients who were diagnosed and underwent radical resections at Peking University Cancer Hospital. Tumor stages were classified based on the 2010 tumor node metastasis (TNM) classification recommended by the American Joint Committee on Cancer (AJCC 7th edition). T and N classifications were evaluated according to the final pathological result, whereas the M classification was determined by surgical and imaging findings. None of the patients received local or systemic treatment prior to surgery. After surgical resection, tissues were sharply frozen via liquid nitrogen and then maintained at -80 °C and prepared for RNA extraction use. Clinic pathological parameters and follow-up information were collected from available patient data. This study was approved by Ethics Committee of Peking University Cancer Hospital (Approval number: 2018KT07). This study was carried out in accordance with the Declaration of Helsinki of the World Medical Association.

### Cell lines

The human GC cell lines (MKN45, MKN28, MGC803, BGC823, SGC7901, N87 and AGS) and normal gastric epithelial cell line GES-1 which were obtained from the Chinese Academy of Sciences (Shanghai, China), and then grown in DMEM (GIBCO, Carlsbad, USA) supplemented with 10% fetal bovine serum (FBS; GIBCO, Carlsbad, USA), and maintained in a 37 °C incubator with a 5% CO_2_.

### RNA extraction and RT-qPCR

Total RNA was extracted from tissue samples and cell lines using TRIzol (Invitrogen, Carlsbad, USA) according to the manufacturer's instructions. First-strand cDNA was generated by reverse transcription polymerase chain reaction using a reverse transcription system kit (Invitrogen, Carlsbad, USA). Reverse transcription-quantitative PCR (RT-qPCR) was performed with the ABI PRISM7500 Sequence Detection System according to the SYBR Green method. For each sample, gene expression was normalized to GAPDH. The primer sequences used in this research were listed in Table S1. The RT-qPCR reactions for each sample were performed in triplicate, and the relative expression level was calculated using the 2^-ΔΔCt^ method.

### Cell Counting Kit-8 (CCK-8) assay

Cells were seeded in a 96-well plate at a density of 3×10^3^ cells/well. On days 1, 2, 3, 5 and 7, the cells were incubated with CCK-8 (KeyGEN biotech, Jiangsu, China) for 3 hrs at 37 °C, and absorbance was measured at 450 nm using a microplate reader.

### Cell transfection

The full-length complementary cDNA of human CXCL16 was synthesized by Invitrogen and cloned into the expression vector pcDNA3.0 (Genechem, China). MGC803 and SGC7901 were transfected with plasmids using Lipofectamine 2000 (Invitrogen, Carlsbad, USA). The small hairpin RNA (shRNA) of the CXCL16 or ADAM10 were provided by Genechem. All knocking down sequences were listed in Supplementary Table S1. Plasmid vectors for transfection were prepared using DNA Miniprep Kits (Tiangen, China), and transfected into GC cells using Lipofectamine 2000, or the lentiviruses encoding shRNAs were used to simultaneously infect GC cells, following the manufacturer's instructions as previously described 20. Empty vector (Con) and non-target shRNA (NT) were applied as controls. Stable cell lines were screened by administration of neomycin or puromycin.

### Colony formation

For the colony formation assay, the established stable cell lines were seeded into 6-well plates at 500 cells/well and incubated at 37 °C with 5% CO_2_ for 14 days. The cells were then washed twice with PBS carefully and fixed with 75% ethyl alcohol for 15 min at room temperature. The cells were stained with 0.1% crystal violet.

### Migration assay and Invasion assay

A migration assay was performed in a Modified Boyden Chamber (Costar, #3422, Cambridge, MA) to examine cell migration. A total of 3×10^4^ cells were suspended in 200 μL serum-free DMEM and seeded onto polycarbonate filters for the migration assay; each lower chamber was filled with 600 μL of 10% FBS-DMEM. For the invasion assay, the top chamber membrane was coated with 40 μl of 0.125 mg/ml matrigel in serum-free DMEM and incubated at 37 °C for 2 hrs before use. To assess the ability of the GC cells to cross the polycarbonate membrane, 5×10^4^ cells in 200 μl of serum-free DMEM were placed into the upper compartment of the wells that were coated with the reconstituted Matrigel, and 600 μl 10% FBS-DMEM was placed into the lower compartment. After 24 hrs of incubation, cells that had migrated or invaded into the lower chamber were fixed for 10 min with 1 ml of 4% formaldehyde (Sinopharm, China) and stained with 0.5% crystal violet for 30 min. After removing the non-migrating cells, the migrated and invaded cells were photographed by an inverted light microscope (magnification, 200×, Nikon Corporation, Japan) at least 6 random fields for each well.

### Western blot analysis

RIPA buffer (R0010, Beijing Solarbio Science & Technology, China) supplemented with protease and phosphatase inhibitors was used to isolate total proteins from treated or untreated pancreatic cancer cells. The proteins were quantified using the bicinchoninic acid kit, separated using 12% sodium dodecyl sulphate-polyacrylamide gel electrophoresis (SDS-PAGE), and transferred onto NC-transfer membrane (HATF00010, Millipore, USA). The NC membranes were blocked with 1% bull serum albumin and incubated with specific primary antibodies at 4 °C overnight followed by the secondary antibody incubation at room temperature for 1 hr. Primary antibodies against N-cadherin, E-cadherin, β-catenin, Snail, Slug, ZO-1, Akt, phospho-Akt, p38, Phospho-p38, Erk, Phospho-Erk were purchased from Cell Signaling Technology. Primary antibodies against CXCL16, CXCR6, and ADAM10 were purchased from Abcam. Mouse anti-GAPDH was purchased from ProteinTech. All primary antibodies were diluted at 1:1000, and the corresponding secondary antibodies were diluted at 1:5000. All antibodies are listed in Supplementary Table S2.

### *In vivo* xenograft mouse model

Animal studies were carried out in strict adherence with institutional guidelines, and approved by the Animal Ethics Committee at Peking University Cancer Hospital (Number: EAEC 2018-22). Female athymic BALB/c nude mice (4-6 weeks, 18-20 g) were used as host mice. The animals were bred in a specific pathogen-free environment at the Laboratory Animal Center of the Peking University Cancer Hospital. A total of 5×10^5^ BGC823 cells stable transfected with shCXCL16 or Non-target control (NT) were subcutaneously injected into the left or right subaxillary, respectively (n=6). Caliper was used to measure the width (W) and length (L) of the xenograft and the volume of xenograft was calculated by the following formula: V=0.5×L×W^2^; when the maximum length of any tumor reached 15 mm or the volume of any tumor reached 800 mm^3^, the experiment was terminated. An electronic balance (Sartorius, BSA224S-CW, Germany) was used to measure the weight of xenografts. The mice were sacrificed 3 weeks after injection, and the xenografts were fixed with formalin and embedded with paraffin. BGC823 cells stable transfected with shCXCL16 or NT (5×10^6^ cells/400 μL volume per mouse) were injected into the BALB/c nude mice via tail vein (n=5). 4 weeks after injection, all the mice were sacrificed and the lungs were collected. Bouin's solution was injected from the main bronchi to fix the lung tissues.

### Immunofluorescence assay

Cells were dispensed into an 8-well chamber slides (LabTek, Thermo Fisher Scientific, USA). For immunofluorescence staining, cells were fixed in 4% paraformaldehyde solution and 0.1% Triton X-100 solution was added to penetrate the cell membrane. After a blocking step, cells were incubated with primary antibody: anti-E-cadherin (dilution 1:400) at 4 °C overnight and secondary antibody Alexa-Fluor-594 conjugated anti-rabbit IgG (dilution 1:400). Hoechst 33342 (Invitrogen, USA) was used as a nuclear counterstain. Samples were analyzed under Zeiss LSM700 confocal laser scanning microscopy (Leica, Germany) equipped with ZEN Zeiss software.

### Immunohistochemistry (IHC)

IHC was performed as previously described 21. Dewaxed in xylene, and washed with graded alcohol for rehydration, the FFPE sections were achieved in 0.01M citrate buffer (pH 6.0) by autoclaving for 3 minutes for antigen retrieval. Then endogenous peroxidase activity was blocked by incubating the slides in 0.3% H_2_O_2_ for 10 min. Primary antibodies against human CXCR6 (dilution 1:500) diluted in DAKO antibody diluent, were applied to sections and incubated in a humidified chamber at room temperature for 1 hour. Antigen visualization was performed with ImmPRESS Peroxidase Polymer Detection Reagents (Vector Laboratories, Japan) and 3,3′-diaminobenzidine (DAB), followed by counterstaining with Mayer's hematoxylin (Sigma-Aldrich, USA). A negative same-species IgG control was included in every experiment. Expression of CXCR6 proteins was evaluated and scored by two independent pathologists under microscopy, who were blind to the patient clinical data. The rate of positive stained cancer cells was evaluated in three randomly selected areas (200×) from the tumor tissue samples. When the average positive tumor rate was >10%, the tumor was defined as being positively stained. Finally, the staining of CXCR6 expression were ascribed to - negative; +, low expression; and ++, high expression.

### Bioinformatics and statistical analysis

RNA-seq data and survival data for patients with GC were downloaded from the Gene Expression Omnibus (GEO) database (GSE22377 and GSE15459) and Gene Expression Profiling Interactive Analysis (GEPIA), an online analysis tool based on The Cancer Genome Atlas (TCGA) and the Genotype-Tissue Expression (GTEx) database. All statistical analyses were performed using SPSS 18.0 for Windows (SPSS, Inc., Chicago, USA) and GraphPad Prism 5.0 software (GraphPad Software, Inc., La Jolla, USA). The significance of Kaplan-Meier statistics was tested using the log-rank test. Multivariate analysis was performed using the multivariate Cox regression model. Relationships between CXCL16 expression and clinicopathological characteristics were analyzed by the Chi-square tests. Multivariate analysis was used to detect the independent prognostic parameters. The differences between groups were analyzed via Student's t-test or one-way ANOVA. Correlations between two variables were assessed using a Pearson's analysis. All experiments were repeated at least three times. Differences were defined as significant as follows: *P<0.05; **P<0.01; ***P<0.001.

## Results

### CXCL16 is upregulated in GC tissues and cell lines

**Figure 1:**
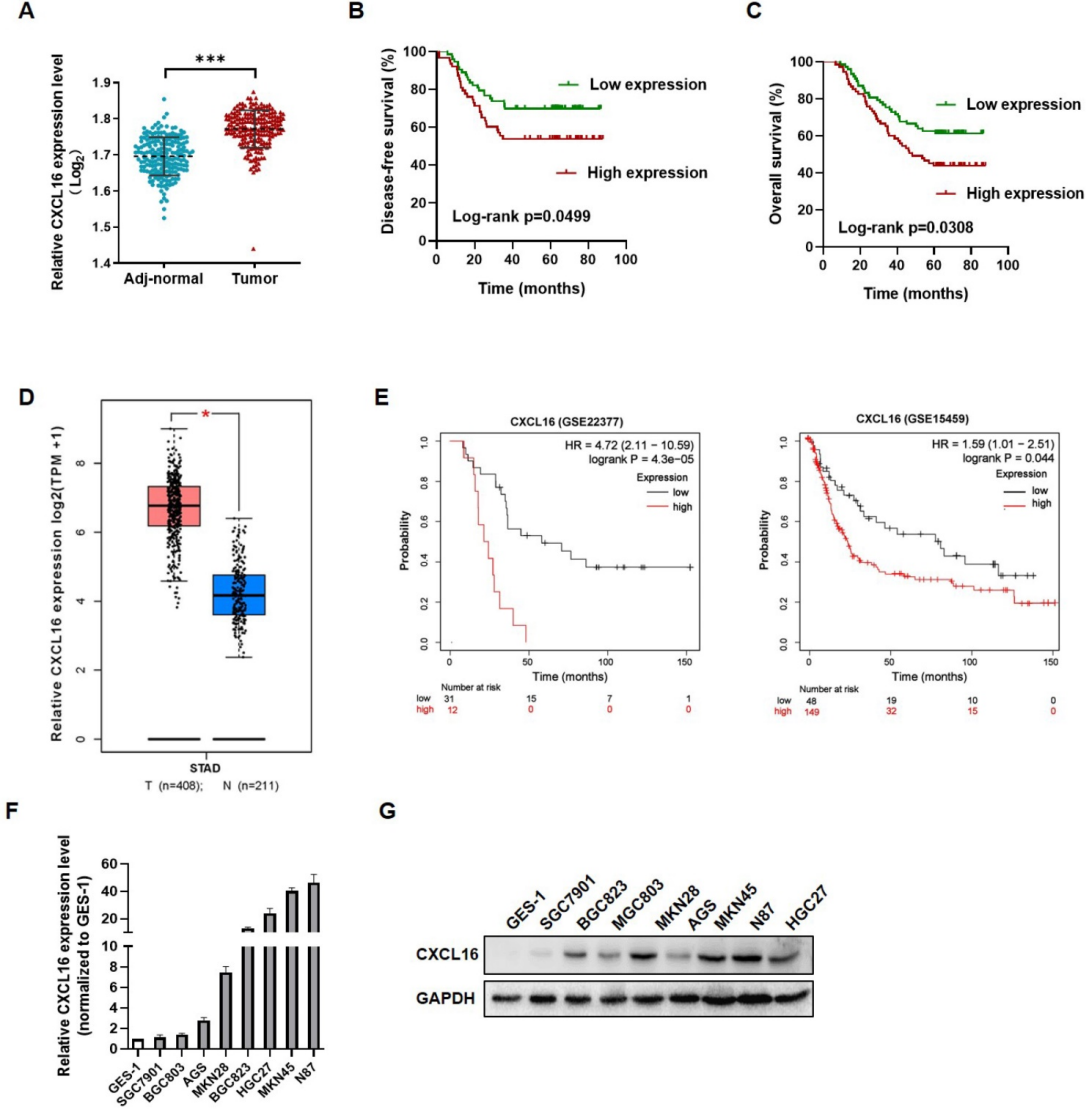
** CXCL16 up-regulated in human gastric cancer tissues and cell lines. (A)** CXCL16 expression was examined by RT-qPCR in GC and adj-normal (n=149).** (B and C)** Kaplan-Meier survival analysis of DFS and OS according to CXCL16 levels in GC patients.** (D)** The expression level of CXCL16 in human GC compared with normal tissues in TCGA database. **(E)** Kaplan-Meier survival analysis of DFS according to CXCL16 mining of public microarray datasets (GSE22377 and GSE15459). **(F and G)** Compared to the cell lines derived from normal gastric mucosa GES-1, the relative expression level of CXCL16 were determined by RT-qPCR or western blot in primary GC cell lines (MKN45, MKN28, MGC803, BGC823, SGC7901, N87 and AGS). Data are presented as the mean ± SD from three independent experiments. *P<0.05, ***P <0.001.

To investigate the role of CXCL16 in GC tumorigenesis, the expression levels of CXCL16 were detected in 149 paired GC tissues and adjacent normal tissues by RT-qPCR. CXCL16 expression was higher in GC tumor tissue compared to the adj-normal tissues (*P*<0.001) (Fig. 1A). Patients were assigned according to their median CXCL16 mRNA level in GC tissues as follows: high-CXCL16 expression group (n=75); low-CXCL16 expression group, (n=74). The clinicopathological parameters of two groups are presented in Table 1. As shown in Table 1, CXCL16 high expression was positively associated with poor differentiation (*P*=0.009) and pTNM stage (*P*=0.003). Multivariate Cox regression analysis of 5-year overall survival (OS) indicated that high expression of CXCL16 was an independent marker for poor prognosis (*P*=0.046) (Table 2). Kaplan-Meier survival curves further demonstrated that disease-free survival (DFS) and OS were worse in GC patients with higher CXCL16 expression than in patients with lower CXCL16 expression, respectively (*P*=0.0499 and 0.0308, Fig. 1B and C). Expression level of CXCL16 was validated using the TCGA database online website GEPIA (http://gepia.cancer-pku.cn/), indicating that CXCL16 significantly upregulated in GC tissue (Fig. 1D) 22. Further, the KM-Plotter online tool was applied to prove that CXCL16 was associated with poor prognosis in two different GC datasets (GSE22377, GSE15459, *P*= 4.3E-05 and 0.044, respectively) (Fig. 1E) 23. We evaluated the expression levels of CXCL16 in GC cell lines by RT-qPCR and western blot. It revealed that CXCL16 was high expressed in GC-derived tumor cell lines compared to the normal gastric mucosa-derived cell line GES-1 (Fig. 1F and G).

### CXCL16 promotes GC cell proliferation, colony formation, migration and invasion

**Figure 2:**
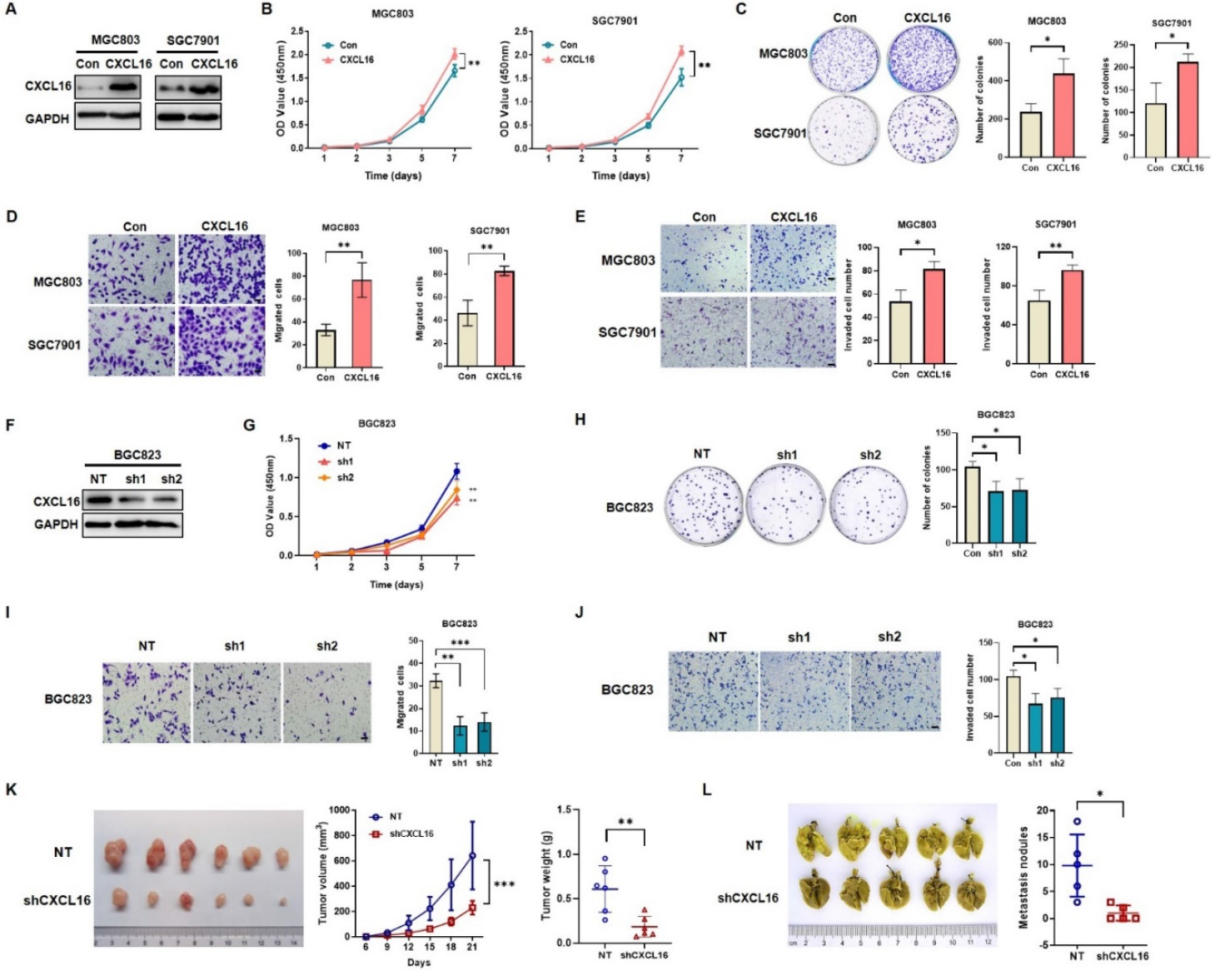
** CXCL16 promotes GC cell proliferation and migration. (A)** The efficiency of CXCL16 overexpression was determined by western blot in MGC803 and SGC7901 cells. **(B-E)** Proliferation assays, colony formation assays, migration assays and invasion assays were preformed to exam the biological function in CXCL16 overexpressed MGC803 and SGC7901 cells. **(F)** The knockdown efficiency of CXCL16 was determined by western blot in BGC823 cell. **(G-J)** Proliferation assays, colony formation assays, migration assays and invasion assays were preformed to exam the knockdown CXCL16 in BGC823 cell. Data are presented as the mean ± SD from three independent experiments. **(K)** The representative images and the quantification of xenograft upon NT or shCXCL16 in BGC823 injected nude mice, respectively (n=6 for each group). Tumor volumes were calculated after injection every 3 days for 21 days. Tumor weights are represented as mean ± SD. **(L)** The representative images and the quantification of lung metastatic colonization of nude mice treated with tail vein injection of BGC823 cells stably transfected with NT or shCXCL16 (n=5 for each group). *P<0.01, **P<0.005, ***P<0.001 *vs.* the control group.

To determine the potential effects of CXCL16 on GC cell growth and cell mobility, we first established stable overexpression CXCL16 in MGC803 and SGC7901 cells; and CXCL16 knock down in BGC823 cell, respectively. Western blot was used to determine the efficacy of transfection (Fig. 2A). *In vitro* results showed that CXCL16 significantly elevated cell proliferation, colony formation, migration and invasion in the overexpression MGC803 and SGC7901 cell lines respectively (Fig. 2B-E). Consistently, CXCL16 depletion inhibited the cell proliferation, colony formation migration and invasion in BGC823 cells (Fig. 2F-J). To further determine the tumorigenic ability of CXCL16 in GC cell lines *in vivo*, xenograft tumor models were established by subcutaneous injection of BGC823 cells with or without CXCL16 depletion. Tumors exhibited a smaller size and slower growth rate in the BGC823-shCXCL16-injected group compared with the BGC823-NT-injected group. These results demonstrated that downregulation of CXCL16 inhibited the tumorigenic ability of GC cells* in vivo* (Fig. 2K). As the high expression level of CXCL16 was associated with distance metastasis in patients with primary GC, we examined the effects of CXCL16 on tumor metastatic colonization. BGC823-shCXCL16 cells or BGC823-NT cells were injected into athymic nude mice via the tail vein. BGC823-shCXCL16-injected mice had fewer lung tumor nodules compared to the BGC823-NT-injected mice (Fig. 2L). Taken together, these data indicated that CXCL16 aggressively promoted cell proliferation migration and invasion in GC.

### CXCL16 promotes EMT via Akt and MAPK signaling pathways

**Figure 3:**
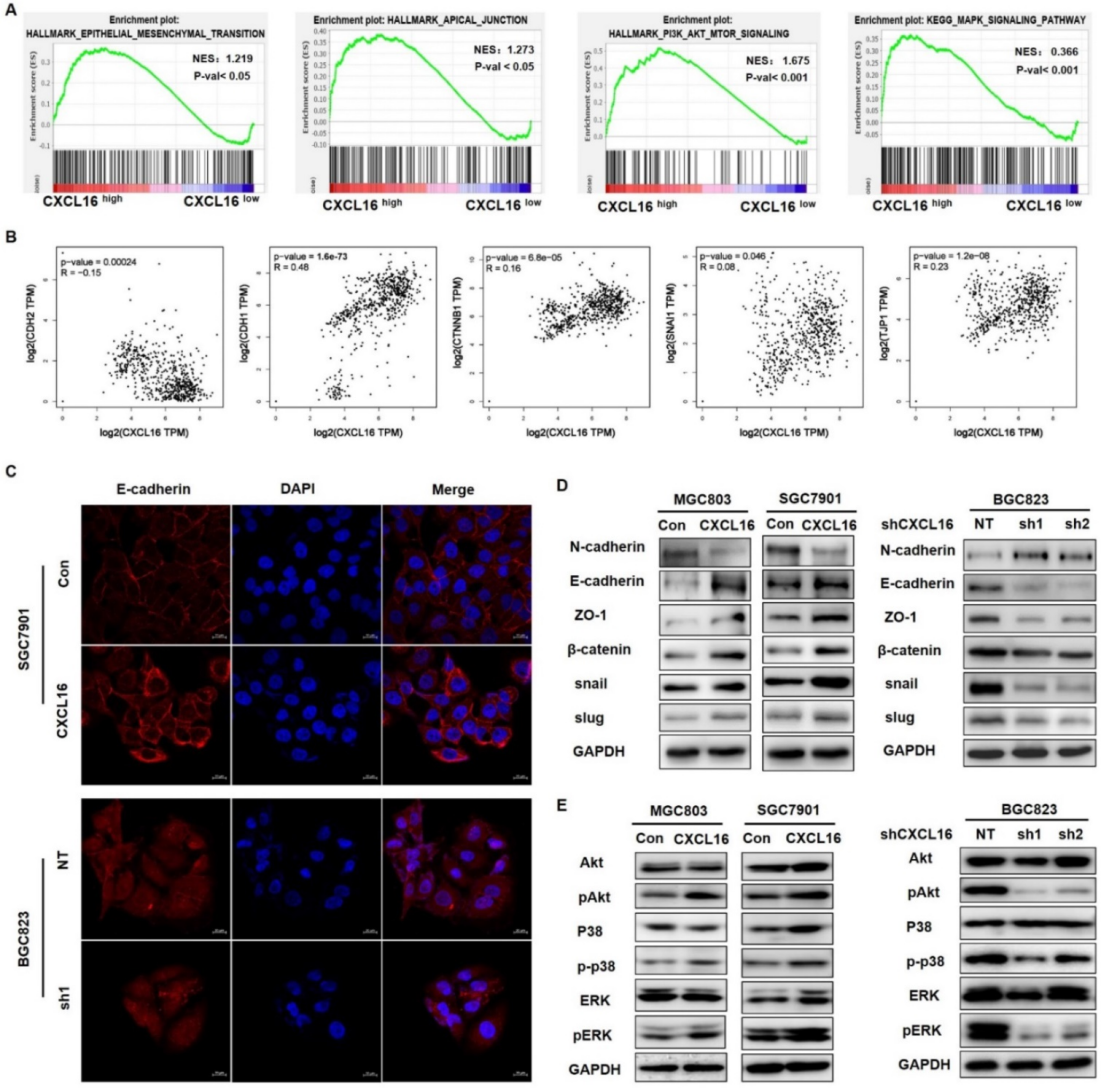
** CXCL16 promotes EMT via Akt and MAPK signaling pathways. (A)** GSEA analysis results of TCGA STAD dataset revealed that HALLMARK EPITHELIAL MESENCHYMAL TRANSITION, HALLMARK APICAL JUNCTION, HALLMARK PI3K-AKT-MTOR SIGNALING and KEGG MAPM SIGNALING PATHWAY were significantly enriched based on CXCL16 high expression. NES, normalized enrichment score; the *P* value indicates the significance of the enrichment score. **(B)** Pearson correlation analysis using TCGA STAD database revealed that CXCL16 expression were significantly correlated with CDH2, CDH1, CTNNB1, SNAIL1, and JPT1 in GC.** (C)** Representative images of immunofluorescence (IF) staining of E-cadherin (red) on CXCL16 overexpression or knockdown GC cell lines.** (D)** The effect of CXCL16 overexpression or knockdown cell lines on the protein levels of EMT markers: N-cadherin, E-cadherin, ZO-1, β-catenin, snail and slug in GC cells.** (E)** The effect of CXCL16 overexpression or knockdown cell lines on Akt and MAPK signaling pathway in GC cells.

To investigate the possible driving mechanism of CXCL16 in promoting tumorigenesis, we used TCGA stomach adenocarcinoma cohort (STAD) to conduct on GSEA. The results revealed that CXCL16 high expression was positively correlated with EMT process, and with Akt and MAPK signaling pathways (Fig. 3A). Pearson correlation analysis in this cohort demonstrated that CXCL16 was significantly correlated with CDH2, CDH1, CTNNB1, SNAIL1, and JPT1, those markers involved in EMT and tumorigenesis (Fig. 3B). Immunofluorescence staining revealed that E-cadherin in the membranes of CXCL16 overexpressed SGC7901 cells was increased, while decreased in the CXCL16 depletion BGC823 cells (Fig. 3C). Western blot further confirmed that overexpression of CXCL16 increased the expression of E-cadherin, ZO-1, β-catenin, snail and slug, a serial number of epithelial biomarker; and decreased the expression of N-cadherin, the biomarker of mesenchymal. While knockdown of CXCL16 significantly decreased the expression of E-cadherin, ZO-1, β-catenin, snail and slug, and decreased the expression of and N-cadherin, indicating that CXCL16 could promote EMT process (Fig. 3D). To further identify the downstream signals pathways of the CXCL16, western blot was used to determine the phosphorylated forms of Erk1/2, p38 MAPK and Akt in GC cells. Overexpression of CXCL16 could activate the Erk1/2, p38 MAPK and Akt pathways, while knockdown of CXCL16 could inactivate those signaling pathways (Fig. 3E). These results suggested an active role for CXCL16 in promoting EMT and activating Akt and MAPK signaling pathways.

### Upregulated CXCL16 promoted CXCR6 and ADAM10 expression in GC

**Figure 4:**
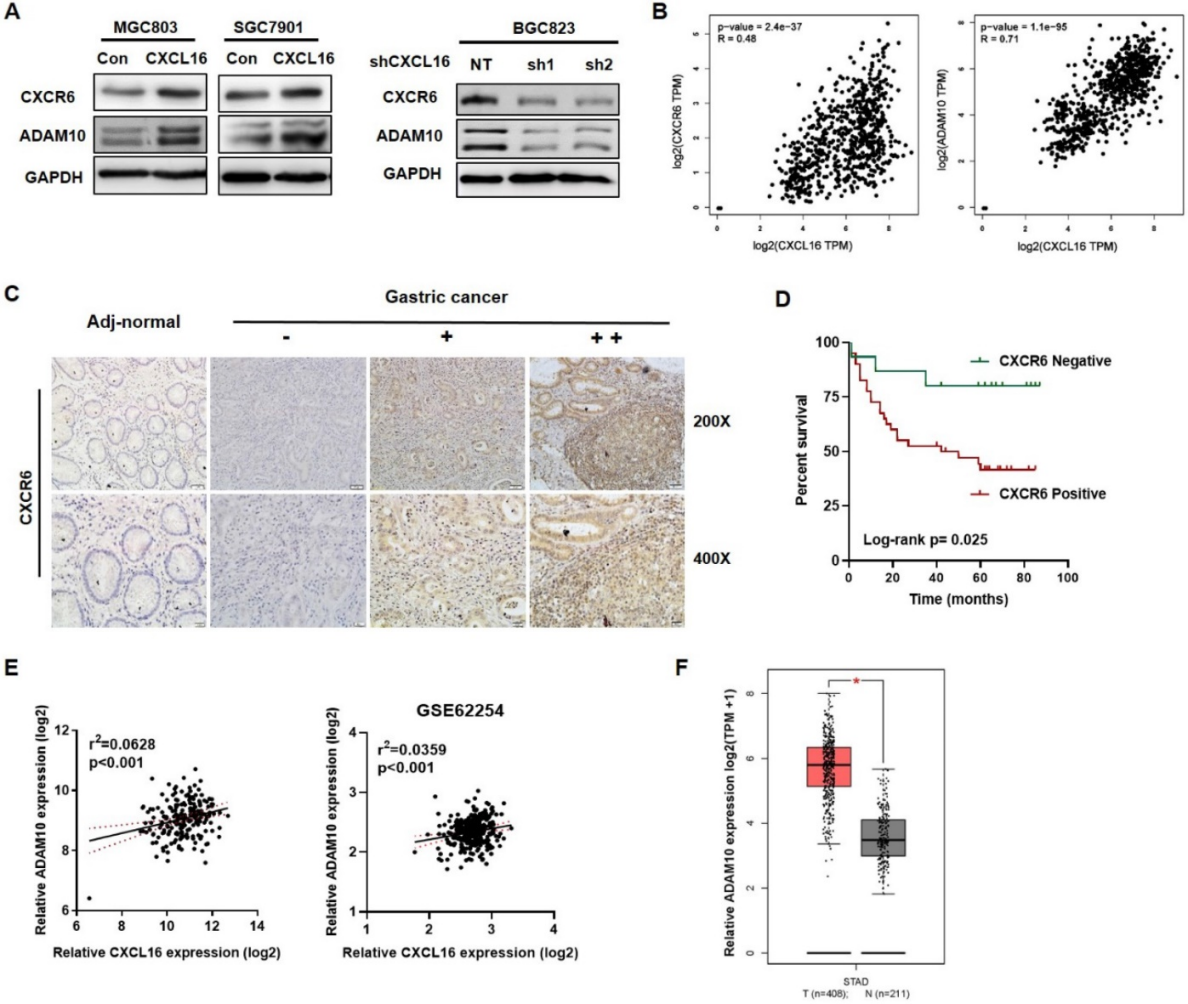
** High CXCL16 was positively correlated with increased CXCR6 and ADAM10 protein expression level in GC. (A)** Verification of CXCR6 and ADAM10 protein expression in CXCL16 overexpressed MGC803 and SGC7901 cells, and CXCL16 knockdown BGC823 cell. **(B)** Pearson correlation analysis using TCGA STAD database revealed that CXCL16 expression were significantly correlated with ADAM10 and CXCR6 in GC. **(C)** Immunohistochemistry, using DAB (brown), showed the protein expression level of CXCR6 in adj-normal tissues and GC tissues: a, negative staining of CXCR6 in adj-normal tissues; negative, weak and high (-, + and ++) staining of CXCR6 in cancer tissues. Magnification, x200 and x400. **(D)** Kaplan‑Meier overall survival curves of patients with GC stratified by negative (n=15) and positive (n=40) expression levels of CXCR6 (*P*=0.025). Patients with positive CXCR6 expression had the poor outcome.** (E)** Pearson correlation analysis confirmed ADAM10 positively relate to the expression of CXCL16 in our previous RNA array dataset (n=198) and GEO public dataset GSE62254 (n=300) respectively. **(F)**The expression level of ADAM10 significantly increased in human gastric cancer compared with normal tissues in TCGA database.

As to the entire CXCL16/CXCR6 axis, CXCL16 would play as a trigger, ADAM10 and CXCR6 would reflect along with CXCL16 expression. We used western blot to determine the protein expression of CXCR6 and the metalloproteases ADAM10 in overexpressed CXCL16 or knockdown CXCL16 GC cell lines. It showed that overexpression of CXCL16 increased the protein level of CXCR6 and ADAM10, knockdown of CXCL16 in the contrary (Fig. 4A). TCGA STAD database revealed that mRNA level of CXCR6 and ADAM10 were positively correlated with the CXCL16 expression (*P*<0.001 and P<0.001) (Fig. 4B). Further, to investigate protein level of CXCR6, the effector in CXCL16/CXCR6 axis, CXCR6 IHC staining was applied. We found that CXCR6 located diffusely in the cytoplasm and cell membrane, and positive expression in 81.8% (45/55), negative in 19.2% (10/55) of GC tissue (n=55), while negative in adj-normal tissues (n=34) (Fig. 4C). Patients were stratified by tumor tissue negative (n=15) and positive (n=40) CXCR6 expression into two groups. The clinicopathologic analysis revealed that levels of CXCR6 was positively correlated with poor differentiation status (*P*=0.034), and depth of invasion (*P*=0.033) (Table S3). Kaplan-Meier survival curves further demonstrated that 5-year OS were worse in GC patients with positive CXCR6 expression than in patients with negative CXCR6 expression (*P*=0.025) (Fig. 4D). Multivariate Cox regression analysis of 5-year OS has not indicated that positive expression of CXCR6 was an independent marker for poor prognosis yet, which may because of less samples (Table S4). On the other hand, as to ADAM10, it was confirmed that in our previous RNA array dataset (n=198) and public dataset from GEO profiles GSE62254 (n=300) respectively, ADAM10 would positively relate to the expression of CXCL16 (*P*<0.001 and *P*<0.001) (Fig. 4E) 19. With TCGA dataset GEPIA validated that ADAM10 itself significantly increased in the tumor tissue than the normal (*P*<0.05) (Fig. 4F). Taken together, as an essential metalloprotease of CXCL16/CXCR6 axis, ADAM10 and the protein level of CXCR6 statistically positive correlation with CXCL16. ADAM10 could positively regulate the CXCL16/CXCR6 axis in GC.

### Knocking down ADAM10 abrogated CXCL16 function in GC cells

**Figure 5:**
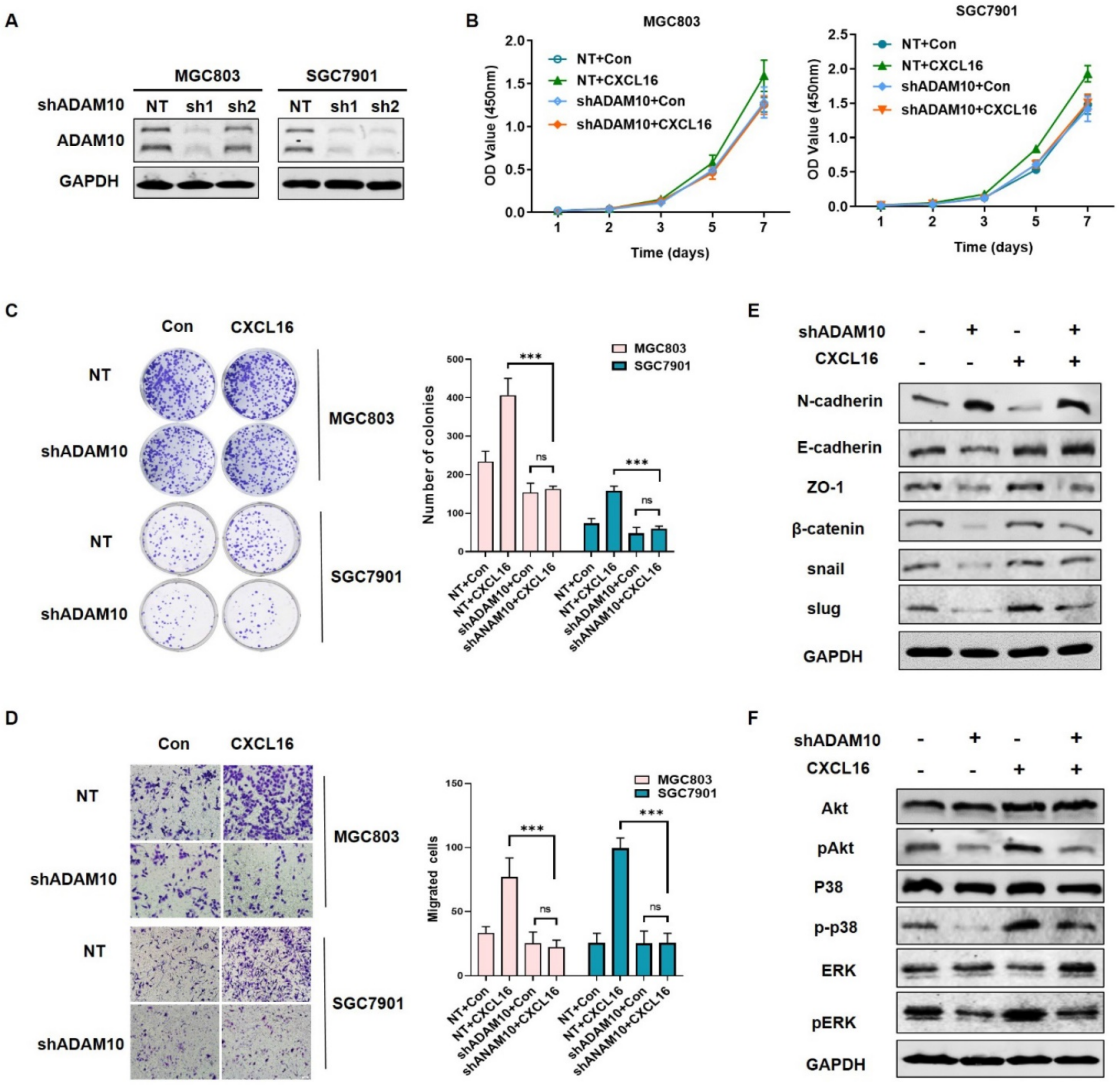
** Knocking down ADAM10 abrogated CXCL16 function in GC cells. (A)** The knockdown efficiency of ADAM10 was determined by western blot in MGC803 and SGC7901 cells, respectively. **(B-D)** Proliferation assays, colony formation assays and migration assay of ADAM10 knockdown cells with or without CXCL16 overexpression in BGC823 and SGC7901 cells respectively. **(E and F)** Western blot was performed to detect the protein levels of EMT markers, Akt and MAPK signaling pathway in ADAM10 knockdown with or without CXCL16 overexpression in SGC7901 cells. *P<0.01, ***P<0.001.

To explored the mechanism of ADAM10 regulating the CXCL16 function in GC, we established ADAM10 knockdown cell lines in MGC803 and SGC7901 respectively, as the ADAM10 knocking down efficiency showed in Fig. 5A. To investigate whether ADAM10 involved in CXCL16 regulation, we compared the proliferation, colony formation and migration ability of the cells overexpressed with CXCL16 in ADAM10 known down cell lines (Fig. 5B-D). It showed that overexpressing CXCL16 in shADAM10 cell could not function as it played in control group, which meant ADAM10 played a critical role in CXCL16 tumorigenic process. Western blot showed that without apparent of ADAM10, overexpressed CXCL16 could not initiate the EMT process and not activate Akt and MAPK cell signaling pathways (Fig. 5E and F). It implied that ADAM10 could abrogated CXCL16 function in GC. These findings indicated that upregulated CXCL16 promoted tumorigenesis in GC cells in a manner depending on the ADAM10 metalloproteases function along with activation of the Akt and MAPK signaling pathways.

## Discussion

GC is one of the major human malignant tumor with a high recurrence rate, and its morbidity and mortality rates are increasing steadily all over the world 24. It is pivotal for GC patients to identify specific prognostic biomarkers and effective drug target. Accumulating evidence indicated that CXC chemokines expressed in tumor were considered as critical factors that interacted in intracellular communication, cell migration, and immune responses that dictated tumor development and progression 25-27. Among these chemokines, CXCL16 has been showed as an important roles in the development of cancers, increasing of tumor infiltrating lymphocytes (TILs), regulating angiogenesis, controlling cell behaviors, and guiding migrating tumor cells to their targeted locations 28. Previous studies have shown that CXCL16 was abnormally expressed in various cancer tissues, and served as markers and promoters for inflammation-associated cancers 12, 29, 30. But the biological function and molecular mechanism of CXCL16 in GC is still unclear. In our previous study, the RNA micro-array screening in GC showed CXCL16 upregulated in tumor tissue. In the present study we have confirmed that CXCL16 was overexpressed in GC by RT-qPCR and significantly correlated with the poor survival of GC patients, which was consistent with TCGA and GEO public dataset. Gain- and loss-of-function experiments were employed to investigate the role of CXCL16 in proliferation and migration both *in vitro* and *in vivo*. It relieved that upregulated CXCL16 would perform as an oncogenic factor.

As CXCL16's orphan ligand, CXCR6, was also reported high expressed in several cancers 31, 32. However, less evidence could explain in the CXCL16/CXCR6 axis signal who is the first trigger factor. Our data showed upregulated CXCL16 associated with CXCR6 accumulation which resulted in poor GC outcomes. The increasing protein level of CXCR6 resulted from abundant of CXCL16. Given that knocking down ADAM10 abrogated the oncogenic biological function of CXCL16 overexpression, we concluded that CXCL16 been cleaved by ADAM10, yielding sCXCL16, initiated CXCR6 activation. After a series of cell signaling transduction, CXCL16 induced the proliferation, migration and invasion of tumor cells. That was how CXCL16 regulated the CXCL16/CXCR6 axis positive feedback loop regulated by ADAM10 in GC. We here mainly focused on the positive feedback of CXCL16 in tumor cell itself. But it was reported that CXCR6 upregulated in a portion of T cells 33, 34, and these CXCR6^+^ T cells would be recruited to the tumor tissue by the gradient of CXCL16. Although higher levels of TILs could be regarded as a favorable prognostic sign, the high expression of CXCL16 in tumor tissues accompanying increasing it self's expression of CXCR6 might effectively interrupted the antitumor immune process. For, tumor CXCL16/CXCR6 axis might dominantly consume the large portion of CXCL16 and promote self-reproduction and migration, less CXCL16 would be released out to recruit the CXCR6^+^ T cells, which might be a distinct tumor escape mechanism in GC.

Our data also showed that knockdown ADAM10 repressed GC tumorigenesis biologically. Because ADAM10 is emerging role as a significant contributor to cancer progression by implicating in the shedding of dozens of substrates that drive cancer progression, such as: Notch, epidermal growth factor (EGF), and ErbB2 35. In our experiment, it was only used as an experimental means to illustrate the mechanism of CXCL16 in GC, and no in-depth exploration was carried out. But ADAM10 as a potential drug target is worthy worth exploring. Previous studies have shown that overexpression of CXCL16 could activate cell signaling pathway, such as: Akt, MAPK, STAT3 or Wnt5a, respectively 32, 36-38. In the present study, mining with TCGA STAD dataset, we found high expression of CXCL16 was positively enriched in EMT genes, PI3K/Akt and MAPK signaling pathway.

In conclusion, we identified CXCL16 was highly expressed in GC tissues and associated with poor prognosis of patients. CXCL16 enhanced GC cell proliferation and migration. Mechanically, with ADAM10's cleavage, upregulated CXCL16 activated the CXCL16/CXCR6 axis, promoted EMT process and regulated PI3K/Akt and MAPK pathways, that led to tumorigenesis. This study revealed the vital significance of CXCL16 in GC progression, implicating the ADAM10-dependent CXCL16/CXCR6 axis and might be a potential prognostic biomarker and therapeutic target in GC.

## Supplementary Material

Supplementary tables.

## Figures and Tables

**Figure 6:**
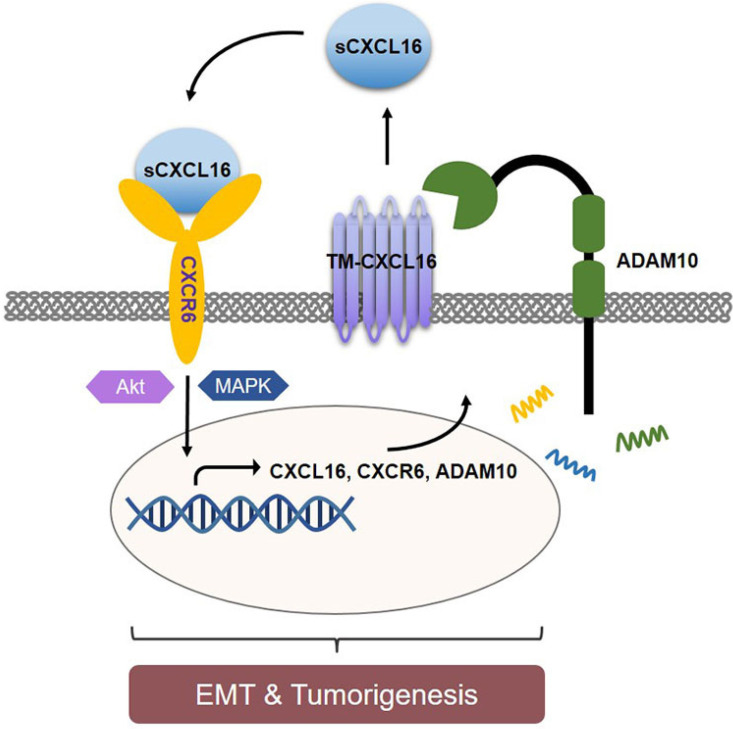
** Schematic diagram representation of the mechanism of CXCL16 promoting tumorigenesis in GC.** Cleaved by ADAM10, upregulation of CXCL16 resulted into accumulation of CXCL16 outside the tumor cells. CXCL16 binding to its orphan receptor, CXCR6, activated the CXCL16/CXCR6 axis which led to the tumorigenesis in GC. And during this process Akt and MAPK signaling pathway were activated.

**Table 1:** Correlation between CXCL16 expression levels and clinicopathological features in patients with gastric cancer

Characteristics	Number (n=149)	Expression of CXCL16		*P*-value
		Low (%)	High (%)	
**Gender**				
Male	116	54 (73.0)	62 (82.7)	0.156
Female	33	20 (23.0)	13 (17.3)	
**Age (y)**				
≤60	87	45 (60.8)	42 (56.0)	0.553
>60	62	29 (39.2)	33 (44.0)	
**Diameter (cm)**				
≤5 cm	73	41 (55.4)	32 (42.7)	0.122
>5 cm	76	33 (44.6)	43 (57.3)	
**Pathological types**				
Adenocarcinoma	110	57 (77.0)	53 (70.7)	0.379
others	39	17 (23.0)	22 (29.3)	
**Histologic differentiation**				
Well or moderate	42	28 (37.8)	14 (18.7)	0.009**
Poor	107	46 (62.2)	61 (81.3)	
**Depth of invasion**				
T1-2	36	23 (31.1)	13 (17.3)	0.051
T3-4	113	51(68.9)	62 (82.7)	
**Lymphatic metastasis**				
No	48	28 (37.8)	20 (26.7)	0.147
Yes	101	46 (62.2)	55 (73.3)	
**Distant metastasis**				
M0	141	73 (98.6)	68 (90.7)	0.031*
M1	8	1 (1.4)	7 (5.4)	
**pTNM stage**				
I-II	78	51 (68.9)	27 (36.0)	0.003**
III-IV	71	23 (31.1)	48 (64.0)	

Note: *P<0.05, **P<0.01.

**Table 2:** Multivariate analysis of prognostic parameters in patients with gastric cancer by Cox regression analysis

Prognostic parameters	Multivariate		*P*-value
	HR	95%CI	
**Gender**			
Male	1.312	0.617-2.782	0.482
Female			
**Age (years)**			
≤60	0.857	0.478-1.535	0.603
>60			
**Histologic differentiation**			
Well or moderate	1.203	0.588-2.460	0.613
Poor			
**TNM Stage**			
I-II	5.206	2.525-10.730	0.000***
III-IV			
**CXCL16 expression**			
Low	2.327	1.016-5.328	0.046*
High			

**Note:** *P<0.05, **P<0.01, ***P<0.001. Abbreviations: HR, hazard ratio; CI, confidence interval.

## Abbreviations

CXCL16: CXC motif chemokine ligand 16
TM-CXCL16: transmembrane CXCL16
sCXCL16: soluble CXCL16
CXCR6: CXC motif chemokine receptor 6
GC: gastric cancer
GSEA: gene set enrichment analysis
ADAM10: a disintegrin and metalloproteases 10
EMT: epithelial mesenchymal transition
STAD: stomach adenocarcinoma cohort
