# CXCL16 Promotes Gastric Cancer Tumorigenesis via ADAM10-Dependent CXCL16/CXCR6 Axis and Activates Akt and MAPK Signaling Pathways: Erratum

**DOI:** 10.7150/ijbs.84342

**Published:** 2023-06-21

**Authors:** Jing Han, Runjia Fu, Cong Chen, Xiaojing Cheng, Ting Guo, Longtao Huangfu, Xiaomei Li, Hong Du, Xiaofang Xing, Jiafu Ji

**Affiliations:** 1 Department of Gastrointestinal Cancer Translational Research Laboratory, Key Laboratory of Carcinogenesis and Translational Research (Ministry of Education), Peking University Cancer Hospital & Beijing Institute For Cancer Research, Fu-Cheng Road, Beijing, China.; 2 Department of Oncology, The Second Hospital, Cheeloo College of Medicine, Shandong University, Jinan, 250033, China.; 3 Department of Gastrointestinal Surgery, Key Laboratory of Carcinogenesis and Translational Research (Ministry of Education), Peking University Cancer Hospital & Institute, Beijing, China.

In our paper, the author noticed errors in Figure 2J and Figure 5D. Representative images of transwell assay (Figure 2J and Figure 5D) were misplaced into the manuscript during the stage of figure preparation. We checked and reanalyzed the original data again and verified that the conclusion of the article was not affected by the errors. In this aspect, all authors have agreed to the erratum, and we do apologize for any inconvenience caused by the negligence in our work.

Figure 2J and Figure 5D should be corrected as follows.

## Figures and Tables

**Figure 2:**
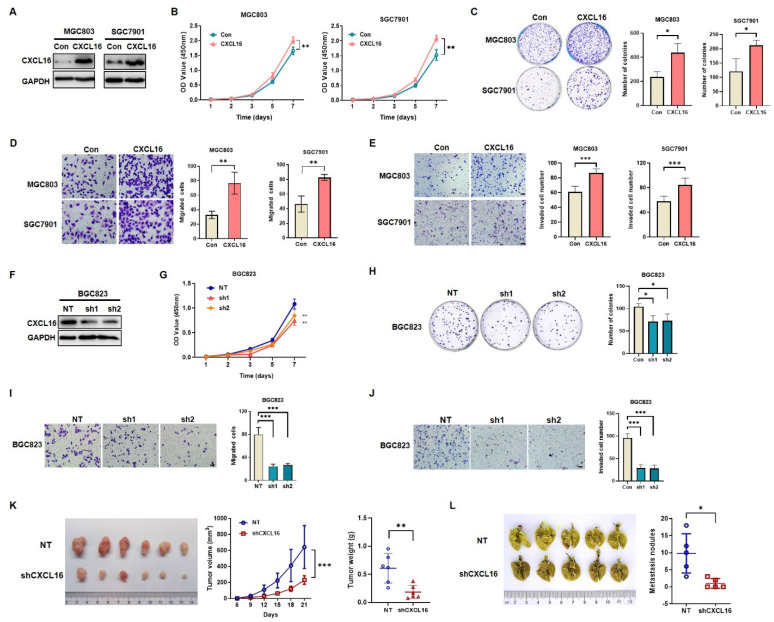
Correct image.

**Figure 5:**
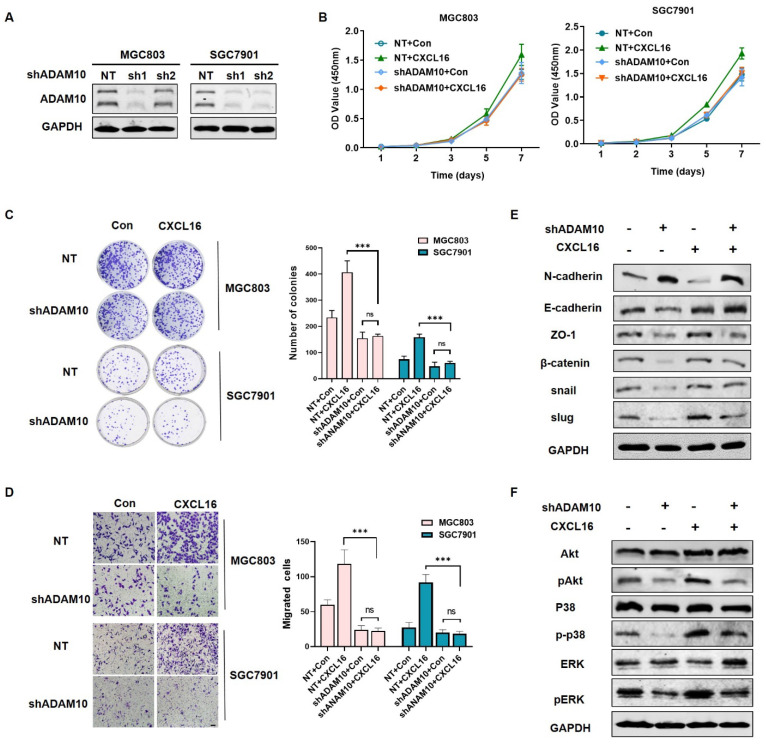
Correct image.

