# Supplementary material for: CXCL16 Promotes Gastric Cancer Tumorigenesis via ADAM10-Dependent CXCL16/CXCR6 Axis and Activates Akt and MAPK Signaling Pathways
Source: Int J Biol Sci. 2021 Jul 5;17(11):2841–52. doi: 10.7150/ijbs.57826 (PMC8326113; doi:10.7150/ijbs.57826)
Supplement: Supplementary file 1 — Supplementary tables. [file ijbsv17p2841s1.pdf]

List of Supporting Information:

Supplementary Table S1. Primers Related to RT-PCR, and Short Hairpin Plasmids Construction.

Supplementary Table S2. Primary antibody used in the study.

Supplementary Table S3. Correlation between CXCR6 IHC expression levels and clinicopathological features in patients with gastric cancer.

Supplementary Table S4. Multivariate analysis of prognostic parameters in patients with gastric cancer by Cox regression analysis.

Supplementary Table S1. Primers related to RT-qPCR, and short hairpin plasmids construction.

Primer set	Primers	Sequence
**RT-qPCR**		
CXCL16	F	5'- AAAGAGCTCACTCGTCCCAA-3'
	R	5'- CAAGCTTCCATTCTTGGCTC-3'
GAPDH	F	5'-GGGCTGCTTTTAACTCTGGT-3'
	R	5'-TGATTTTGGAGGGATCTCGC-3'
**Knocking down target sequence**		
CXCL16	sh1	5'- CUCACUCGUCCCAAUGAAATT-3'
	sh2	5'- GGAUCACUGUCCUCGGACATT-3'
ADDAM10	sh1	5'- GGGTCTGTTATTGATGGAAGA-3'
	sh2	5'- GCTGATGAGAA GGACCCTACA-3'

Supplementary Table S2. Primary antibody used in the study.

Primary antibody	Catalog	Company
β-Catenin(D10A8)	#8480S	Cell Signaling Technology
E-Cadherin	#3195	Cell Signaling Technology
N-Cadherin	#13116	Cell Signaling Technology
Phospho-p38 MAPK	#8690S	Cell Signaling Technology
Phospho-p38 MAPK (Thr180/Tyr182)	#9211S	Cell Signaling Technology
p44/MAPK (Erk1/2)	#4695S	Cell Signaling Technology
Phospho-p44/42 MAPK (Erk1/2) (Thr202/Tyr204)	#9101	Cell Signaling Technology
Akt (pan) (C67E7)	#9272	Cell Signaling Technology
Phospho-Akt (Ser473)	#4060	Cell Signaling Technology
Slug	#9585T	Cell Signaling Technology
Snail (C15D3)	#3879	Cell Signaling Technology
ZO-1	#5406	Cell Signaling Technology
CXCL16	ab101404	Abcam
ADAM10	ab1997	Abcam
CXCR6	ab8023	Abcam
GAPDH	60004-1-IG	Proteintech

Supplementary Table S3. Correlation between CXCR6 IHC expression levels and clinicopathological features in patients with gastric cancer.

Characteristics	Number (n=55)	Expression of CXCR6		*P* -value
		Negative (%)	Positive (%)	
Gander				
Male	36	8 (53.5)	29 (72.5)	0.068
Female	19	7 (46.7)	11 (27.5)	
Age (y)				
≤60	32	9 (60.0)	23 (57.5)	0.729
>60	23	6 (40.0)	17 (42.5)	
Diameter (cm)				
≤5cm	35	10 (66.7)	25 (67.6)	0.950
>5cm	17	5 (33.3)	12 (32.4)	
Pathological types				
Adenocarcinoma	45	12 (80.0)	33 (82.5)	0.832
others	10	3 (20.0)	7 (17.5)	
Histologic differentiation				
Well or moderate	45	15(100.0)	30 (75.0)	**0.034***
Poor	10	0 (0.0)	10 (25.0)	
Depth of invasion				
T1-2	14	7 (46.7)	7 (17.9)	**0.033***
T3-4	40	8 (53.3)	32 (82.1)	
Lymphatic metastasis				
No	13	4 (26.7)	9 (22.5)	0.748
Yes	42	11 (73.3)	31 (77.5)	
Distant metastasis				
M0	51	15 (100.0)	36 (92.3)	0.274
M1	3	0 (0.0)	3 (7.7)	
pTNM stage				
I-II	15	6 (40.0)	9 (22.5)	0.198
III-IV	40	9 (60.0)	31 (77.5)	

Note: *P<0.05.

Supplementary Table S4. Multivariate analysis of prognostic parameters in patients with gastric cancer by Cox regression analysis.

**Prognostic parameters**	**Multivariate analysis**		***P*-value**
	**HR**	**95%CI**	
Gander			
Male<br>Female	2.219	0.818-6.020	0.118
Age (years)			
≤60<br>>60	3.284	1.281-8.418	**0.013***
Histologic differentiation			
Well or moderate<br>Poor	0.185	0.039-0.887	**0.035***
CXCR6 expression			
Negative<br>Positive	1.945	0.550-6.872	0.302

Note: *P<0.05.

Abbreviations: HR, hazard ratio; CI, confidence interval.
